# Guidelines for interprofessional practice in rehabilitation at primary health care level

**DOI:** 10.4102/phcfm.v17i1.5079

**Published:** 2025-10-27

**Authors:** Luzaan Africa, Jose Frantz, Nondwe B. Mlenzana

**Affiliations:** 1 Interprofessional Education Unit, Faculty of Community and Health Sciences, University of the Western Cape, Bellville, South Africa; 2 Office of the Deputy Vice-Chancelor: Research and Innovation, University of the Western Cape, Bellville, South Africa; 3 Department of Physiotherapy, Faculty of Community and Health Sciences, University of the Western Cape, Bellville, South Africa

**Keywords:** interprofessional practice, primary health care, rehabilitation, clinical practice guidelines, interprofessional education, delphi technique

## Abstract

**Background:**

Interprofessional practice (IPP) is essential for strengthening rehabilitation services within primary health care (PHC) settings. However, many healthcare professionals currently in the workforce have not been trained in interprofessional education (IPE), which limits effective collaboration.

**Aim:**

This study aimed to develop and validate interprofessional activity guidelines that align with core interprofessional competencies and support the implementation of a rehabilitation model at the PHC level in South Africa.

**Setting:**

The study was conducted remotely with geographically diverse experts but remained grounded in the South African PHC context. It focused specifically on the Western Cape Department of Health.

**Methods:**

A two-round Delphi technique was used to gather expert consensus. In Round One, 15 experts identified 26 interprofessional activity guidelines aligned with the 5 phases of an existing PHC rehabilitation model. In Round Two, 11 experts evaluated the guidelines for consensus and convergence. A consensus threshold of 70% agreement and a convergence threshold of a median score above 3.24 were used.

**Results:**

Of the 26 guidelines, 25 achieved the required 70% consensus. One guideline, which did not reach the consensus percentage, was retained based on a median score above 3.24, indicating convergence of expert opinion. All guidelines were mapped to the four IPE core competencies.

**Conclusion:**

This study presents validated interprofessional activity guidelines to enhance rehabilitation services at the PHC level. Aligned with core competencies, these guidelines support practical implementation through a phased approach, with readiness assessments and ongoing evaluation recommended. The Delphi-informed process may be adapted for similar resource-limited health systems.

**Contribution:**

The results from this study provides validated activity guidelines that translate a South African health policy vision into actionable steps for IPP in the rehabilitation sector at the PHC level. The guidelines strengthen teamwork, communication and patient-centred care across disciplines, offering a replicable model for improving coordination and service delivery in African PHC contexts.

## Introduction

The Quintuple Aim for healthcare improvement outlines five interconnected goals to guide health system reform.^1^ These aims include improving population health outcomes, enhancing patient experience, ensuring health service providers’ well-being, advancing health equity and reducing healthcare costs.^1^ Interprofessional Education and Collaborative Practice (IPECP) is an innovative approach to achieving the quintuple aim.^2^ Given this benefit, interprofessional education (IPE) has been integrated into health professions education. Interprofessional education is when students from two or more disciplines learn with, from and about each other to advance effective collaboration towards improved patient health outcomes.^3^ However, with it being a contemporary approach to education, many health professionals practising within healthcare settings may not have been trained within an IPE-informed curriculum,^4^ thus necessitating a concerted effort to develop principles and guidelines for interprofessional practice (IPP) to ensure that the current health workforce can adapt to the calls for collaborative models of care.

**FIGURE 1:**
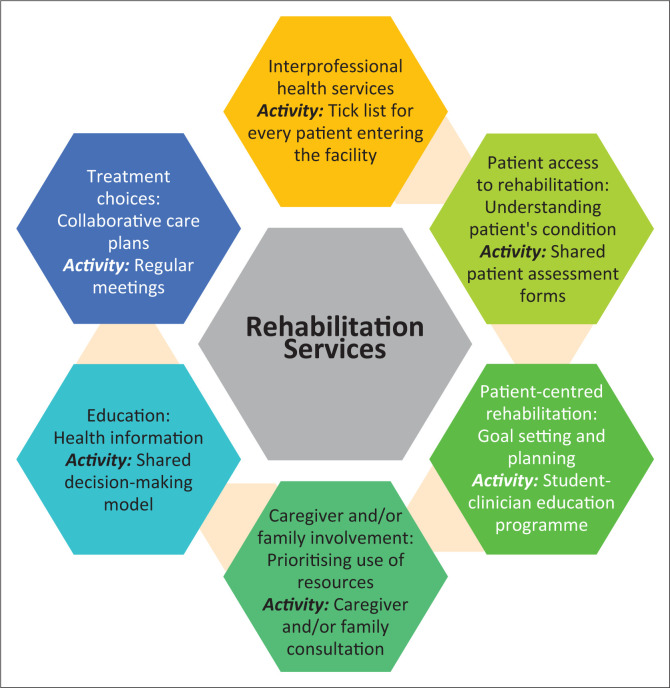
The activities that promote interprofessional practice at phases of an existing rehabilitation model.

In the Western Cape province of South Africa, the Department of Health introduced the Healthcare 2030 policy to improve the health system performance through the provision of accessible, acceptable and patient-centred care at all levels of care, including at the primary health care (PHC) level.^5^ Although the policy outlines broad goals, it fails to provide a clear method to operationalise these goals, particularly in the rehabilitation sector. In response, a rehabilitation model was developed to conceptualise the Healthcare 2030 policy into the PHC sector.^6^ However, while outlining five phases, the model does not offer specific activities or strategies for implementing these phases. In response, a systematic review identified the activities that advance IPP at each phase of the rehabilitation model (Figure 1).^6^

Reviews of literature revealed how IPP activities have been incorporated into clinical practice to improve patient outcomes and provider collaboration.^7,8^ Yet, in South Africa, barriers to implementation remain, including limited theoretical understanding, role confusion and a general lack of awareness among providers about what IPP entails.^9^ Without concrete guidelines, the translation of the Healthcare 2030 policy through the lens of IPP may be compromised. Clinical practice guidelines can bridge this gap by offering context-sensitive, evidence-based recommendations to guide health service providers.^10,11,12,13,14^ Locally developed guidelines are especially important, as PHC settings in South Africa face unique challenges, including hierarchical structures, logistical constraints and limited infrastructure.^9^ It is suggested that, because of increasing international research and knowledge, local researchers should update, adopt, adapt and implement practice guidelines.^15^ Thus, it is important to explore how the training of health service providers should be approached. The IPE core competencies influence the development of health professions education curricula and the training of qualified health professionals.^16^ The IPE core competencies are defined as the skills, knowledge and attitudes health professionals need to collaborate effectively.^16^ With an overarching domain of interprofessional collaboration, there are four competency domains, namely interprofessional communication (CC), teams and teamwork (TT), values and ethics (VE) and roles and responsibilities (RR).^16^

Each of the four IPE competency domains comprises a set of sub-competencies,^16^ which form the foundation of interprofessional learning for lifelong learning.^17^ This illustrates the importance of using the IPE core competencies when developing training opportunities for health professionals. If the Western Cape Department of Health aims to achieve the goals of the Healthcare 2030 policy, health service providers require actionable recommendations on how to adapt their practice to meet the goals of this policy. This study, therefore, had a dual aim: to develop consensus-based IPP activity guidelines using the Delphi technique and to map the guidelines to the interprofessional core competencies. This dual focus ensures that the guidelines assist health professionals in translating policy into practice in a way that is contextually relevant and internationally aligned.

## Research methods and design

### Research design

This study employed a Delphi technique, an iterative method used to obtain expert consensus from specialists in areas where evidence is limited or emerging.^18^ The Delphi design was appropriate for this study as it allowed for input from a diverse panel of experts on the development and feasibility of IPP activity guidelines in rehabilitation sectors at the PHC level. Each round of the Delphi was informed by the data collected in the preceding round.

### Setting and study context

This study was conducted remotely via online questionnaires, allowing participation regardless of geographical location. Despite the variety of participants, the focus of the study was grounded in the South African PHC context. To ensure contextually relevant services for the districts within the nine provinces in South Africa, the district health system allowed provinces to manage their health service delivery.^19^ However, the lack of structural integration of separately functioning health departments resulted in underperformance of the PHC method in certain regions of the country. This study focuses on one province’s health department, the Western Cape Department of Health, which comprises six of the 52 national health districts.^20^

### Population and sampling

According to Hsu and Stanford,^18^ there is no specific criterion for participants, but participants are chosen based on their experience and background related to the topic. As the sustainability of IPP relies on the synergy between health workforce planning and health professions education,^2^ it is important to involve participants from a diverse range of expertise. Participants were purposively recruited based on their knowledge and expertise in PHC, rehabilitation, health professions education and IPP. Invitations were sent to 20 experts identified through professional networks, including the Africa Interprofessional Education Network and the Southern African Association for Health Educationalists and prior collaborative work with IPE. The first round of the Delphi was completed by 15 experts (75% response rate). In the second round, 11 of the original 15 participants responded, indicating a 26.7% attrition rate between rounds.

### Data collection procedure

The data collection procedure was conducted in two rounds.

#### Round one

Participants who provided a positive response to the initial email were sent a link to a Google Form, which included a compulsory consent section at the start of the form. The form was divided into sections informed by the five phases of an existing rehabilitation model.^6^ Each phase of the rehabilitation model was presented together with the proposed activity identified in a related systematic review.^8^ Questions focused on identifying how these interprofessional activities could be implemented within the South African PHC rehabilitation settings to achieve the various phases.

#### Round two

Responses from Round One were analysed to extract guidelines for the implementation of the proposed activities at each phase. Based on these, a 5-point Likert scale questionnaire was developed, ranging from ‘Strongly Disagree’ (1) to ‘Strongly Agree’ (5). Participants were then invited to complete the second round using a second Google Form link.

### Data analysis

According to Hsu and Stanford,^18^ researchers need to find a suitable method to analyse the qualitative information. Open-ended responses from Round One were thematically analysed^21^ with the phases of the rehabilitation model serving as the deductive themes. Notes were made on each response, and themes were synthesised into draft guideline statements.

For Round Two, descriptive statistics were used to analyse the Likert scale data. The 5-point Likert scale was dichotomised into three categories: ‘non-consensus’, which comprises the ‘Strongly disagree’ and ‘Disagree’ ratings; ‘consensus’, which comprises the ‘Strongly agree’ and ‘Agree’ ratings; and ‘Not sure’. The following measures were applied to determine consensus and convergence: consensus was defined as ≥ 70% of participants selecting ‘Agree’ or ‘Strongly Agree’,^18^ while a median rating of ≥ 3.24 was considered to demonstrate convergence of opinion.^22^ A median of 3.24 or higher measures central tendency and best indicates the convergence of opinion among a panel of participants.^18,22^ Given the small sample size, complex statistical tests were deemed unnecessary.

### Ethical considerations

Ethical clearance to conduct this study was obtained from the University of the Western Cape BioMedical Research Ethics Committee (No. BM19/1/38).

## Results

### Characteristics of participants

Table 1 summarises their professional backgrounds, areas of expertise and years of experience of the participating experts. Most participants were members of the Southern African Association of Health Educationalists. Participants could select more than one area of expertise, and many did. The three available categories were IPE, PHC and rehabilitation. Most participants had over five years of experience and a diverse mix across these three fields, contributing to the depth of the Delphi process.

**TABLE 1:** Characteristics of participants.

Characteristic	Category	Number
Profession	Interprofessional education lecturer	1
	Nursing	3
	Occupational therapy	3
	Health professions education	1
	Psychology	3
	Physiotherapy	2
	Doctor	2
Area of expertise	Interprofessional education	11
	Primary health care	11
	Rehabilitation	5
Years of experience	Less than 5 years	2
	5–10 years	5
	More than 10 years	8

### Guidelines for interprofessional activities

The findings are presented according to the six key activity areas that correspond with the phases of the rehabilitation model.^6^ In Round One, experts generated 26 activity-specific guidelines (Table 2). In Round Two, these guidelines were rated using a 5-point Likert scale to determine consensus. Guidelines were considered to have reached consensus if at least 70% of responses fell into the ‘Agree’ or ‘Strongly Agree’ categories. A median score above 3.24 was used to indicate convergence of opinion.

**TABLE 2:** Guidelines for interprofessional activities.

Guidelines	Range	Consensus	Median
**Guidelines for tick list of services**			
1. Interprofessional discussions	3–5	91	4.6
2. All staff must develop the tick list	4–5	100	4.6
3. Tick list must be written in a culturally appropriate language style	3–5	82	4.4
4. List all the services at the facility	3–5	91	4.6
5. Highlight the medical needs of the patient	4–5	100	4.8
**Guidelines for shared patient assessment forms**			
1. Discuss roles of represented staff	4–5	100	4.8
2. Identifies overlapping roles	2–5	82	4.6
3. All staff must develop the form	3–5	91	4.6
4. Avoid discipline-specific jargon	4–5	100	4.5
5. Captures patient’s current condition and progress	4–5	100	4.9
**Guidelines for student-clinician education programme**			
1. Collaborate with local higher education institutions	2–5	82	4.3
2. Higher education institutions to place students in IP teams	4–5	100	4.6
3. Weekly student presentations on specific patient cases	3–5	91	4.5
4. Student-clinician discussions to develop collaborative care plans	4–5	100	4.7
**Guidelines for caregiver and/or family consultations**			
1. Decide on conditions to include	2–5	64	3.6
2. Involve rehab care worker (if no family member present)	4–5	100	4.5
3. Space for family and/or caregiver feedback in patient folder	4–5	100	4.7
**Guidelines for shared decision-making**			
1. Choose the virtual meeting space (e.g. apps)	3–5	82	3.9
2. Develop SDM stationery	3–5	91	4.5
3. Develop collaborative goals for patients	4–5	100	4.9
4. Include patients in decision-making	4–5	100	4.9
5. Include SDM on the tick list to show it has been carried out	4–5	100	4.91
**Guidelines for regular meetings**			
1. Set dates and times in advance	3–5	91	4.6
2. Decide on virtual or face-to-face meetings	4–5	100	4.8
3. Develop role clarification pamphlets	3–5	91	4.6
4. Collaborate with staff according to roles	3–5	91	4.6

IP, interprofessional; SDM, shared decision-making.

#### Tick list of services for each patient entering a primary health care facility at the first point of contact

Experts strongly supported the use of a tick list to document available services for every patient entering the facility. This activity was considered a reminder to create a shared understanding of patient needs and available interprofessional services. All five guidelines reached high levels of consensus, with consensus rates ranging from 82% to 100% and median scores between 4.4 and 4.8. Experts emphasised that tick lists should reflect both medical and psychosocial dimensions of care and be co-developed by all staff in culturally appropriate language.

#### Shared patient assessment form

Participants agreed that shared patient assessment forms enhance communication and coordination by providing a comprehensive overview of the patient’s condition. All five guidelines received ≥ 82% consensus, with median scores between 4.5 and 4.9. Experts recommended avoiding discipline-specific jargon and including overlapping roles in the tool design. The International Classification of Functioning, Disability and Health framework was suggested as a starting point.

#### Student-clinician education programme

The inclusion of students in interprofessional teams was seen as a key strategy for cultivating collaborative behaviours in professional development. All four guidelines achieved consensus, with two receiving 100% agreement. Experts highlighted the importance of collaborating with higher education institutions, suggesting structured placements and weekly student presentations as strategies to foster integration.

#### Caregiver and/or family consultations

Caregiver involvement was viewed as essential for continuity of care and for contextualising patient needs. Two of the three proposed guidelines reached consensus. The third guideline, ‘Decide on conditions to include’, fell short (64% agreement) but had a median above the convergence threshold (3.6) and was therefore retained. Experts stressed that even in the absence of family, rehabilitation care workers should be involved to ensure community-based insights are not lost.

#### Shared decision-making

This activity was strongly endorsed as a mechanism for engaging patients meaningfully in their care. Experts noted that patients must be educated on their diagnosis and prognosis to contribute meaningfully to shared decisions. All five guidelines received consensus with high median scores (3.9–4.9), including the use of collaborative goal-setting and decision-making tools. The inclusion of shared decision-making on the tick list was also highlighted to ensure visibility and accountability.

#### Regular meetings

Regular, focused meetings were considered critical for maintaining collaboration and addressing emerging patient needs. All four guidelines reached ≥ 91% consensus, with median scores from 4.6 to 4.8. Participants encouraged flexibility in using virtual or in-person formats but stressed the need for role clarification pamphlets and pre-scheduled dates to maximise attendance and relevance. Some experts suggested that case managers be included to facilitate continuity, while others warned that overly rigid structures might hinder flexibility.

### Summary of consensus

Across the guidelines for all activities, 25 out of 26 guidelines met the 70% consensus threshold. One guideline in the caregiver consultation domain did not reach full consensus but was retained based on convergence (median > 3.24). All guidelines had median ratings above 3.24, indicating general agreement among experts.

These findings suggest strong expert support for a structured interprofessional approach to rehabilitation services at the PHC level, grounded in shared tools, patient involvement and collaborative education.

## Discussion

This study developed and refined 26 IPP activity guidelines to support the operationalisation of the rehabilitation model at the PHC level in South Africa. Through expert consensus using a Delphi approach, the study aligned these guidelines to the four interprofessional core competency domains proposed by the Interprofessional Education Collaborative (IPEC): VE, RR, CC, and TT.

The findings offer a structured, context-specific response to the implementation gap in South Africa’s Healthcare 2030 strategy, particularly in PHC rehabilitation services where fragmented care, weak collaboration and uni-professional silos remain challenges.

### Integration with Interprofessional Education Collaborative core competencies

**FIGURE 2:**
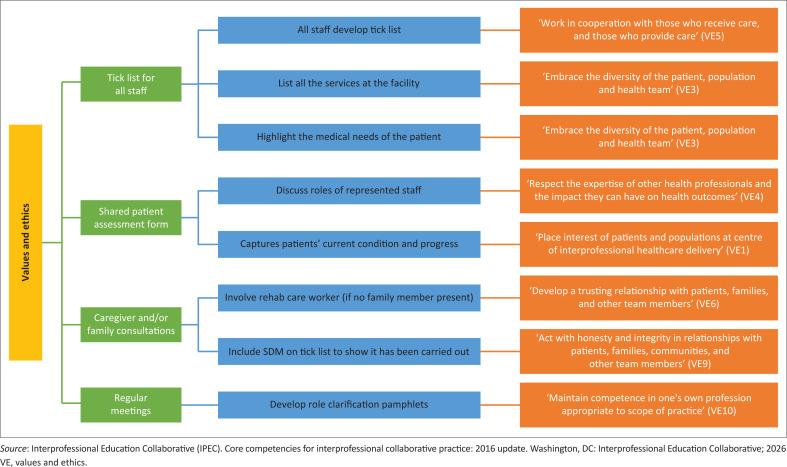
Guidelines that promote values and ethics interprofessional core competency.

A central contribution of this study lies in demonstrating how locally developed practice guidelines can support international interprofessional standards. Each guideline was mapped to one or more IPEC domains. Figure 2 to Figure 5 illustrate how the guidelines developed intersect with the VE, RR, CC and TT domains.

**FIGURE 3:**
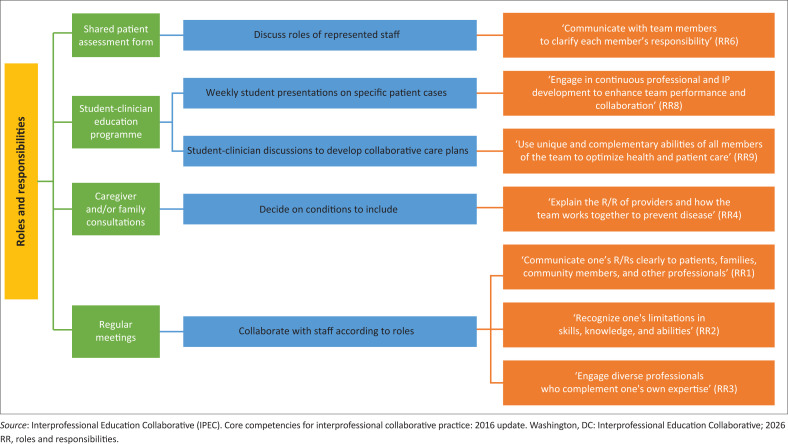
Guidelines that promote roles and responsibilities interprofessional core competency.

**FIGURE 4:**
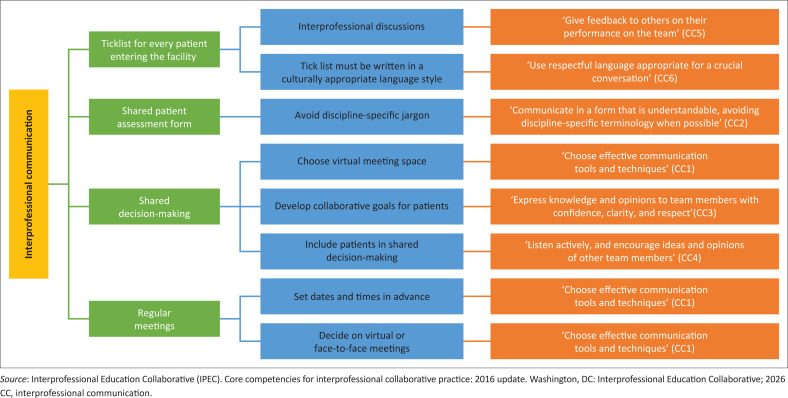
Guidelines that promote interprofessional communication core competency.

**FIGURE 5:**
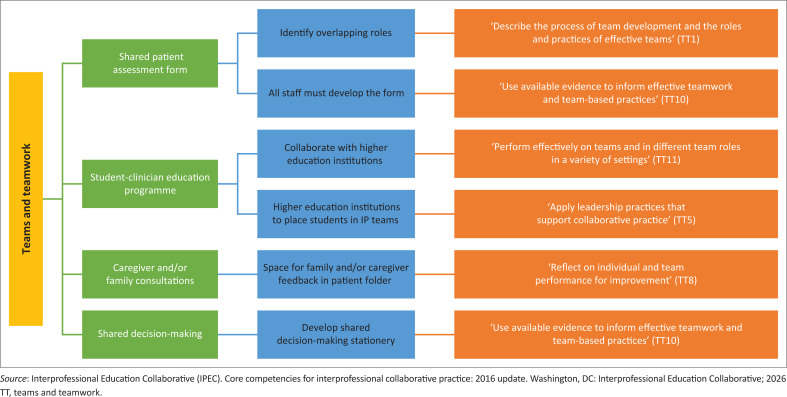
Guidelines that promote teams and teamwork interprofessional core competency.

#### Values and ethics

The VE core competency domain expects health service providers to work with individuals from different professions while maintaining respect and upholding shared values.^16,17^ This domain has 10 sub-competencies that health professionals are expected to demonstrate.^16,17^ In this Delphi study, seven of the 26 guidelines developed by the experts developed or enhanced the VE core competency (Figure 2).

In practice, the successful implementation of interprofessional ethics develops a shared goal of patient, client and community-centred quality care among health professionals.^23^ Health service providers at a PHC facility were resistant to the implementation of IPP at their PHC because of a lack of patient follow-up in their current practice.^9^ However, the development of the values and ethics core competency could encourage healthcare teams to develop shared patient goals, which means that despite follow-up from one discipline, the patient’s goals could still be achieved.

#### Roles and responsibilities

The RR domain is the ability of a professional to use knowledge of their own role and the role of individuals from different professional backgrounds to assess and manage the health needs of a patient.^16,17^ This domain is composed of 10 sub-competencies, which service providers are required to demonstrate in practice. Seven of the guidelines developed by the experts in this study matched the sub-competencies in the RR domain (Figure 3).

The adoption of these guidelines into practice has the potential to strengthen the RR core competency among health service providers. In practice, enhanced clarity around professional roles can assist providers in meeting patients’ health needs more efficiently, reducing duplication of services and enabling better workload distribution.^24^ For example, shared care planning outlined in the guidelines can ensure that each team member contributes within their scope, avoiding unnecessary repetition of interventions. This, in turn, helps maximise the use of health service providers’ time.^24^ One of the reasons for the lack of implementation of IPP at a South African PHC facility is time constraints.^9^ By offering structured, role-clarifying activities and clear collaborative pathways, these guidelines may reduce inefficiencies and directly address time constraints.

#### Interprofessional communication

The interprofessional communication domain emphasises the ability of service providers to communicate with all stakeholders in the health and other sectors to support health promotion, disease prevention and treatment through teamwork.^16,17^ This domain is upheld by eight sub-competencies related to communication. In this study, experts highlighted six guidelines that aligned with the sub-competencies in this domain (Figure 4).

Poor communication in healthcare may result in delayed patient management, misdiagnoses, increased medical errors or patient death.^25^ Generally, health professionals acknowledge the interprofessional differences among team members, including the diversity in health professions education, language barriers and poor role clarity.^25^ However, challenges to interprofessional communication persist in practice.^25^ In a South African PHC facility, logistics and infrastructural barriers hinder successful communication between health service providers.^9^ The incorporation of the proposed guidelines provides clear recommendations on how the sub-competencies of the CC core competency domain can be achieved. These guidelines offer solutions to challenges faced at the PHC facility and therefore provide a strategy for improving interprofessional communication among the health service providers.

#### Teams and teamwork

The final competency domain is TT, which is described as the health professional’s ability to apply values and principles of relationship-building and teamwork to plan, provide and evaluate patient-centred care in a collaborative manner.^16,17^ This domain is composed of 11 sub-competencies, six of which have been covered in the guidelines developed by the experts in this study (Figure 5).

Effective teamwork among health service providers, patients or clients, families and communities avoids gaps in treatment and redundancies and increases effectiveness and efficiency.^24^ The incorporation of these guidelines into clinical practice could develop or enhance the teamwork core competency among health service providers and therefore improve the effectiveness of the services rendered to patients. Several guidelines, such as the shared patient assessment form, align with three or more competency domains. This reinforces their role as structural enablers of interprofessionalism: tools that simultaneously promote communication, teamwork, ethics and role clarity.

### Implementation considerations

This study mainly focused on the development and validation of guidelines to promote IPP in rehabilitation services at the PHC level. While the guidelines were developed through expert consensus, their successful implementation depends on adaptation to local contexts, including institutional support, availability of local resources and the lived realities of health professionals at that facility. These guidelines are aligned with the rehabilitation model that addresses multiple factors of service delivery. As such, we recommend a phased implementation approach, prioritising the guidelines that respond to the most urgent needs of a given facility. As a result of human resource shortages at the PHC level, some facilities lack certain disciplines,^26^ which may limit full implementation. It is therefore imperative to consider context-specific challenges, inefficiencies and resource limitations when applying these guidelines. A related study demonstrates how specific IPP activities can be used to address specific problems in the rehabilitation sector at the PHC level.^27^ Building on this, this article suggests that facilities identify the challenges most relevant to their setting and implement the corresponding guidelines that respond directly to those needs.

Prior to implementation, it is recommended that a readiness assessment for IPE be conducted. The Readiness for Interprofessional Learning Scale (RIPLS) is one of the most widely used tools for this purpose, although it has also been critiqued in IPE literature.^28,29,30^ Rather than discarding the tool in its entirety, scholars suggest that adaptations and refinements are necessary to ensure it remains a valid measure of student readiness, especially as disuse would neglect empirical evidence.^28^ The RIPLS is used to assess the extent to which learners are prepared to move beyond siloed education and engage in a multi-professional learning environment.^31^

In contrast, the Interprofessional Collaborative Competency Attainment Scale (ICCAS) does not measure readiness but assesses self-perceived changes in interprofessional collaborative competencies.^32^ Typically administered before and after IPE training interventions,^32^ the ICCAS has been used in global IPE initiatives to evaluate students’ development across the IPE core competencies.^33^ Together, tools like the RIPLS and ICCAS can be used in monitoring and evaluating both the preparedness of participants and the outcomes of IPP activities in the rehabilitation sector at the PHC level.

### Strengths and limitations

While the guidelines were developed through expert consensus, they have not yet been piloted or evaluated in practice. The Delphi method prioritises agreement, which may suppress dissenting or context-specific concerns. Additionally, the guidelines developed in this study did not align with all the sub-competencies under each domain. However, this study creates the platform to initiate the transition to an interprofessional model of healthcare. Upon implementation of the guidelines, health service providers may identify additional guidelines.

### Implications of the study

To fully realise the potential of the IPP guidelines developed in this study, several strategic steps are recommended. Firstly, pilot implementation at selected PHC facilities should be pursued, accompanied by structured feedback mechanisms involving healthcare providers, PHC managers, and patients to assess feasibility and impact. Secondly, integrating the guidelines into in-service training and continuing professional development initiatives can further enhance interprofessional collaboration among existing staff. Additionally or finally, academic–clinical partnerships should be strengthened to support curriculum alignment and student placements that reflect these interprofessional principles.

To address concerns around time constraints in PHC, integration with existing services delivery platforms and continuous professional development programmes can help ease the implementation burden on clinicians. Future research should focus on evaluating the implementation process and outcomes of these guidelines in real-world settings. In particular, the RIPLS and ICCAS may be valuable for monitoring both readiness and changes in IPE core competencies. Importantly, the Delphi-informed approach used in this study may serve as a replicable model for developing context-sensitive guidelines in other resource-limited or decentralised health systems seeking to embed IPP into healthcare.

## Conclusion

This study offers a blueprint for embedding IPP into PHC rehabilitation in South Africa. Through a Delphi-informed process, 26 expert-validated activity guidelines were developed to address known systemic barriers, including role confusion, poor coordination of care and time constraints. Aligned with global interprofessional competency frameworks, these guidelines provide actionable steps towards collaborative healthcare. If adopted with institutional support and evaluated through readiness and competency tools, they could shift interprofessionalism from policy aspiration to action. Moreover, this model offers a replicable approach for other resource-constrained health systems striving to realise the quintuple aim of health and the vision of Healthcare 2030.
